# Transforming growth factor-β signaling in systemic sclerosis

**DOI:** 10.7555/JBR.31.20170034

**Published:** 2018-01-26

**Authors:** Nolan B. Ayers, Chen-Ming Sun, Shi-You Chen

**Affiliations:** Department of Physiology & Pharmacology, University of Georgia, Athens, GA 30602, USA.; Department of Physiology & Pharmacology, University of Georgia, Athens, GA 30602, USA.; Department of Physiology & Pharmacology, University of Georgia, Athens, GA 30602, USA.

**Keywords:** systemic sclerosis, transforming growth factor-β, mechanism, therapeutics

## Abstract

Systemic sclerosis (SSc) is a complex, multiorgan autoimmune disease of unknown etiology. Manifestation of the disease results from an interaction of three key pathologic features including irregularities of the antigen-specific immune system and the non-specific immune system, resulting in autoantibody production, vascular endothelial activation of small blood vessels, and tissue fibrosis as a result of fibroblast dysfunction. Given the heterogeneity of clinical presentation of the disease, a lack of universal models has impeded adequate testing of potential therapies for SSc. Regardless, recent research has elucidated the roles of various ubiquitous molecular mechanisms that contribute to the clinical manifestation of the disease. Transforming growth factor β (TGF-β) has been identified as a regulator of pathological fibrogenesis in SSc. Various processes, including cell growth, apoptosis, cell differentiation, and extracellular matrix synthesis are regulated by TGF-β, a type of cytokine secreted by macrophages and many other cell types. Understanding the essential role TGF-β pathways play in the pathology of systemic sclerosis could provide a potential outlet for treatment and a better understanding of this severe disease.

## Introduction

Systemic sclerosis (SSc) is a relatively rare disease with prevalence estimates ranging from 30 to 443 per 1 million adults^[1]^. SSc primarily affects middle-aged women but also impacts children and men of all ages^[2]^. Although rare, SSc is a deadly disease in which more than half of the patients diagnosed die due to the damage to internal organs^[3]^. Even though characteristics of the disease have been well documented, the pathogenesis of SSc remains largely unknown. Fibrosis of the skin due to an accumulation of collagen is a common presenting finding of the disease, and the extent of skin involvement is one way in which SSc is classified^[4]^. The two main subsets are limited cutaneous subset (lcSSc) and diffuse cutaneous subset (dcSSc) based on the extent of skin involvement of the disease^[2]^. In limited cutaneous subset skin thickening is limited to the distal part of the extremities, and a systemic involvement is minimal; whereas in diffuse cutaneous subset systemic involvement is prominent, and skin thickening is widespread^[5]^. To allow for earlier, more precise diagnosis of the disease, the American College of Rheumatology (ACR) and the European League Against Rheumatism (EULAR) have developed new classification criteria for SSc^[6– 7]^. According to the new standards, skin thickening of the fingers extending proximal to the metacarpophalangeal joints is sufficient for classifying a patient with SSc^[6]^. Also, seven additive items are given a numerical weight: skin thickening, fingertip lesions, telangiectasia, abnormal nailfold capillaries, lung involvement, Raynaud’s phenomenon, and SSc-related autoantibodies^[6, 8]^.

## The pathogenesis of systemic sclerosis

Systemic sclerosis is characterized as an autoimmune rheumatic disease^[9]^. Although the exact etiology of the disease remains unknown, immunological activation, vasculopathy, and collagen accumulation are three major features of SSc, which seem to be inter-related in a manner that is yet to be fully understood^[10]^. Vascular endothelial activation and proliferative vasculopathy are related to the immunoinflammatory abnormalities associated with the production of autoantibodies^[11]^. Immunological irregularities are a central feature of the disease, as evidenced by early autoantibody production, with antinuclear antibodies being detected in more than 90% of patients with SSc^[12]^. It has also been discovered that B cells have multiple functions in the disease development including autoimmune activation^[12– 14]^. An overexpression of CD19, a critical regulator of B cell activation, is observed in naïve and memory B cells of patients with SSc^[13– 14]^. There is also a significant elevation in the levels of various autoantibodies in transgenic mice overexpressing CD19^[14]^. This finding suggests that the increase in CD19 expression may induce autoantibody production in humans with SSc^[13– 16]^. Therefore, abnormal B cell activation, which augments the cytokine production and consequently tissue fibrosis, could connect immunoinflammatory abnormalities with fibrosis, a characteristic of SSc^[13, 15]^.

Another potential link between immune activation and fibrosis characteristic of SSc could be found in innate immune response. Accumulation of inflammatory infiltrates such as macrophages is common in early stages of the disease, and the increased expression of certain cytokines is known to activate macrophages toward either an M1 or M2 phenotype^[17]^. M1 macrophages encourage inflammatory response and are generally activated by interferon-gamma (IFN g)^[15– 16]^. On the other hand, anti-inflammatory M2 macrophages are important for wound repair and vascularization and are activated by interleukin 4 (IL-4) or IL-13^[15– 16, 18]^.

Toll-like receptors (TLRs) play critical roles in linking immune activation with fibrosis characteristic of SSc. TLRs are a class of pattern recognition receptors (PRRs) expressed on membranes of macrophages, fibroblasts, and various other cell types^[19– 20]^. TLR signaling is mediated by a cytoplasmic Toll/IL-1 receptor (TIR) domain-containing adaptor, MyD88, that recruits IL-1 receptor-associated kinase-4 (IRAK4). IRAK4 is then activated *via* phosphorylation and is associated with TNF receptor associated factor-6. This leads to activation of I kB kinase complex, MAP kinases (JNK, p38 MAPK), and nuclear factor- kB^[21– 22]^. TLRs function to recognize conserved pathogenic-associated molecular patterns (PAMPs) from invasive moieties^[19– 20]^. In addition to PAMPs, endogenous ligands known as damage-associated molecular patterns (DAMPs) also activate TLR signal transduction pathways. DAMPs are released as a result of tissue injury, and their activation of TLR pathways results in the production of cytokines and inflammatory mediators^[19, 23– 25]^. For example, upregulation of TLR2 in SSc leads to an increase in secretion of pro-inflammatory cytokine IL-6 as a response to the endogenous ligand amyloid A, which is a marker of inflammation in patients with the disease^[26– 27]^. Endogenous ligands for TLR4 are released in response to cellular damage, oxidative stress, and extracellular matrix (ECM) remodeling, which also contribute to pathological fibrosis in SSc^[21]^. In fact, constitutive expression of TLR4 in skin and lung fibroblasts of patients with SSc can result in overactive collagen synthesis as well as an enhanced sensitivity to TGF-β1 stimulation^[19, 21, 24]^. Due to their significant roles, a better understanding of the mediators of TLR signaling pathways could assist in explicating potential therapeutic targets to treat SSc^[19, 28– 29]^.

In addition to immunological activation, vasculopathy plays a key role in the pathogenesis of SSc^[5, 30– 31]^. The etiology of early vascular damage in SSc is uncertain, but immunological dysfunction appears to play a role, and could result from cytotoxic T cells, or auto-antibodies targeting microvascular endothelial cells^[5, 32– 33]^. Although vasculopathy predominately affects small and medium-sized arteries, digital ulcers and dilatation of the nailfold capillaries have been noted in the earliest stages of the disease followed by the loss of capillaries later in the course of the disease^[32, 34]^. This damaged capillary architecture in patients with SSc can lead to increased expression of vascular endothelial growth factor (VEGF), stimulating angiogenesis^[35– 37]^. Due to the complexity of generating new capillaries, angiogenesis is mediated by multiple signaling pathways, and deregulation of those pathways can result in dysfunctional capillary formation^[37– 39]^. VEGF and TGF-β are two key mediators that may contribute to defective angiogenesis in SSc^[5, 38]^. In general, VEGF initiates angiogenic sprouting, and TGF-β plays a fundamental role in regulating cell migration, proliferation, and matrix synthesis^[38– 39]^. Furthermore, TGF-β signaling can be either pro- or anti-angiogenic, depending on which pathway is activated^[5, 38– 39]^. Plasma levels and expression of both VEGF and TGF-β are elevated in skin of patients with SSc, along with heightened levels of other proangiogenic mediators^[5, 37]^. As aforementioned, this can result from the damaged capillary architecture that is common at early stages of SSc.

An abnormal balance of vasoconstrictors such as Endothelin-1 (ET-1) and vasodilators such as nitric oxide (NO) also contributes to vascular dysfunction in patients with SSc, with increased expression of ET-1 in the lungs, kidneys, blood vessels, and skin of patients with the disease, and decreased release of NO from vascular endothelium in patients^[5, 32, 35, 40]^. ET-1 is mainly produced by endothelial cells and mediates multiple fibrotic responses including smooth muscle cell proliferation, and vasoconstriction^[41]^. Two types of receptors for ET-1 (ETα and ETβ) are expressed by vascular smooth muscle cells and endothelial cells respectively^[5, 40– 41]^. ETα receptors can mediate vasoconstriction, and pro-inflammatory responses, while ETβ receptors mediate vasodilation *via* the release of NO^[42]^. Correlation between increased levels of ET-1 and several clinical manifestations of SSc, including the development of digital ulcers, suggests that ET-1 plays a role in both vasculopathy and fibrosis characteristic of SSc^[5, 41, 43]^. Additionally, NO release from vascular endothelium and the expression of endothelial NO synthase (eNOS) are decreased in patients with SSc, further contributing to vasculopathy^[5, 40]^. While NO reduces synthesis of ET-1, growth factors such as TGF-β induce synthesis^[5, 40]^. TGF-β may also regulate metabolism of NO, further exacerbating endothelial cell activation^[40]^. Due to the variety of pro-inflammatory effects mediated by ET-1 and its regulators, targeting of its receptors and pathways is promising in managing the disease^[42]^. For example, Bosentan, a dual endothelin receptor antagonist used to treat pulmonary artery hypertension (PAH), acts as a competitive inhibitor of ET-1 decreasing vascular resistance^[41– 42]^. Molecules like Bosentan that block ET-1 receptors are also used for the prevention of digital ulcers in patients with SSc^[41– 42]^. While treatment for vascular components of SSc have become well established in the past few years, successful therapeutics for fibrosis have yet to come to fruition^[33– 34]^.

Endothelial cell abnormalities that affect microvasculature cause an increase in the release of various chemokines, cytokines, and growth factors resulting in the activation of myofibroblasts that are responsible for tissue fibrosis and proliferative vasculopathy characteristic of SSc^[5, 31, 44]^. Increased serum and tissue levels of TGF-β1 have been observed in patients with SSc suggesting its role in the pathogenesis of fibrosis associated with the disease^[44– 47]^. While it is well known that TGF-β is a central mediator of fibrosis, its function in the vascular pathology of SSc is yet to be fully understood^[44– 48]^. Several recent studies have implicated TGF-β signaling as a mediator of PAH with increased levels of TGF-β signaling in the lungs of models with PAH, and blocking of its signaling has been shown to reduce the severity of experimental PAH^[49– 50]^. It's pleiotropic functions in inflammation, fibrosis, and vascular remodeling suggest that TGF-β signaling could play a central role as a link between fibrosis and vasculopathy characteristic of SSc.

## Animal models for systemic sclerosis

Due to inherent challenges of studying SSc in patients, many inducible and genetic animal models have been developed for the study of initial events, genes, and other influences on manifestation of the disease^[9, 46, 51]^. Despite the multitude of animal models that simulate selective aspects of SSc, a lack of models encompassing the disease’s full clinical heterogeneity has hindered the development of successful therapies^[9, 31, 46]^. For example, tight skin 1 mice (*Tsk1*^+/+^) with a homozygous lethal mutation that causes thickened skin firmly bound to the subcutaneous tissue have proven useful for studying the efficacy of drugs that target fibrosis, but the model does not address the etiology of SSc^[51]^. Many animal models effectively display the pro-fibrotic features of SSc without reflecting the vascular characteristics that frequently precede fibrosis^[9, 47, 52]^. For this reason, new genetic animal models have been developed in order to reproduce the pathophysiological cascades of SSc that lead to its key features, rather than only mimicking selective features. For example, the Fos-related antigen-2 (FRA-2) transgenic mouse model expresses key features of the disease that result from abnormalities of vasculature^[51, 53]^. Both microangiopathy and progressive skin fibrosis are displayed in FRA-2 transgenic mice^[51]^. The FRA-2 gene belongs to the activator protein-1 (AP-1) family of transcriptional regulators that control a multitude of downstream effects in response to cellular stress^[51, 53]^. Cell proliferation, apoptosis, and inflammation are all regulated by the AP-1 family^[51]^. The FRA-2 transgenic mouse model allows for the study of fundamental characteristics of SSc development, and might serve as a preclinical model for prospective therapeutics^[51, 53]^.

Another animal model utilizes mice with double heterozygous deficiency of two transcription factors, Friend leukemia integration 1 (Fli1) and Krüppel-like factor 5 (KLF5), to mimic the epigenetic phenotype of SSc skin^[54]^. Fli1, a member of the E26 transformation-specific family, plays a role in activation, differentiation, and development of fibroblasts, endothelial cells, and immune cells^[55]^. Fli1 mediates a non-canonical pathway of TGF-β and represses the type I collagen gene, but is epigenetically downregulated in dermal fibroblasts of patients with SSc^[54– 55]^. Similarly, gene expression of KLF5, a member of the SP/KLF transcription factor family that plays a role in cell proliferation, is downregulated in SSc skin^[54, 56]^. Mice with double heterozygous deficiency of KLF5 and Fli1 display all three key pathological features of SSc, including fibrosis, vasculopathy, and autoimmunity^[9, 54]^. This model allows for a more holistic study of the mechanisms involved in the pathophysiology of SSc. For example, it has been suggested that canonical and non-canonical pathways of TGF-β are activated in Fli1^+/−^; KLF5^+/−^ skin as a result of induced phosphorylation of Smad3, ERK, p38, and JNK by heterozygous Fli1 and KLF5^[54]^.

In addition to genetic models that attempt to mimic the pathological events leading to SSc, inducible models also exist and prove beneficial for studying target molecules and the effects of potential therapeutics on further development of significant features of the disease^[9, 51, 57]^. Bleomycin-induced skin and lung fibrosis is among the most established models commonly used to mimic inflammatory and fibrotic changes that occur early in the course of SSc development^[9]^. Upon intravenous, subcutaneous, or intraperitoneal administration of bleomycin, levels of proinflammatory cytokines increase, followed by a peak expression of growth factors such as TGF-β1 around day 20^[58]^. Bleomycin-induced fibrosis is commonly used due to accessibility and the ease of reproduction, as well as its mimicry of inflammatory changes that are characteristic of early development of SSc^[58]^. However, limitations of the traditional bleomycin model separate the bleomycin-induced fibrosis model from the actual pathogenesis of SSc. For example, in humans with the disease, microangiopathy generally precedes development of fibrosis but does not occur in the bleomycin model^[58– 59]^. Additionally, fibrosis due to the traditional bleomycin model only develops at the site of injection while fibrosis in patients with SSc is systemic, affecting internal organs as well as the skin^[51, 58– 59]^. Recently, modified murine models for administering bleomycin have been developed that aim to more accurately represent the systemic inflammation that is characteristic of SSc^[59]^. With the use of implanted osmotic minipumps in the skin of mice, bleomycin can be continuously infused over a period of one to four weeks as opposed to daily subcutaneous injections^[59]^. This model mimics human SSc more accurately with both skin and lung fibrosis, as well as more stable dermal inflammation^[59]^.

Other inducible models attempt to recreate the induction of tissue fibrosis and collagen accumulation with the use of reactive oxygen species (ROS), which are produced in large amounts in skin fibroblasts of patients with SSc^[9, 60]^. Inducible models that use ROS generally aim to recreate the induction of tissue fibrosis, with less of a focus on vasculopathy and immunological facets of the disease^[9]^. With that being said, daily subcutaneous injections of hypochlorous acid (HOCl) to BALB/c mice over a period of six weeks generates various ROS and induces both localized and lung fibrosis, as well as the production of anti-DNA topoisomerase-1 auto-antibodies that are characteristic of dcSSc^[61]^. Additionally, ex vivo analysis of the lungs of HOCl-treated mice shows evidence of inflammatory infiltrates- mostly T cells^[61]^. Another inducible animal model recreates similar features of SSc with subcutaneous injections of topoisomerase-1 and complete Freund’s adjuvant (CFA)^[62]^. This model reproduces main characteristics of SSc including anti-topoisomerase antibodies, increased levels of pro-inflammatory cytokines IL-6, and IL-17, as well as increased levels of TGF-β1 and a decrease in anti-inflammatory cytokine, IL-10^[62]^.

With animal models that closely represent the full clinical heterogeneity of SSc, a better understanding of the molecular mechanisms that contribute to its manifestation will follow. On the other hand, as the relationships between various facets of SSc are elucidated, more encompassing models may be developed, which in turn will lead to new insights into the disease mechanisms.

## TGF-β signaling in systemic sclerosis

TGF-β is a key fibrogenic cytokine that regulates a multitude of biological functions including cell proliferation, differentiation, apoptosis, tissue homeostasis and regeneration^[45, 63– 64]^. Due to its diversified activities, malfunctions in TGF-β-related processes can lead to severe, multifaceted diseases. The extent of TGF-β involvement in vasculopathy characteristic of SSc remains relatively unknown, but its homeostatic functions in both endothelial cells and vascular smooth muscle cells (VSMCs), as well as its role in tissue fibrosis, have made TGF-β an attractive target for multiple drug development^[44, 48, 65– 68]^.

The TGF-β superfamily consists of almost 30 proteins in mammals, including TGF-β, growth/differentiation factors (GDFs), activins and inhibins, and bone morphogenetic proteins (BMPs)^[65]^. TGF-β signaling regulates gene expression mainly *via* activation of Smad transcription factors, although other non-canonical pathways also exist^[63]^. The activation of the Smad pathway involves a TGF-β superfamily ligand binding to a heterodimer of receptors, including TGF-β type I and type II receptor (TβRI and TβRII)^[63, 65]^. The TGF-β type II receptor is a serine/threonine receptor kinase that upon activation causes the phosphorylation of a type I receptor^[65]^. Phosphorylation of TβRI initiates the phosphorylation of the Smad2 or 3 protein, which then forms a Smad complex with a Smad4 protein^[45, 63– 65]^. This activated Smad complex then enters the nucleus of a cell where it acts as a transcription factor^[12]^. Through the Smad3 mechanism, TGF-β1, the most abundant of three TGF-β isoforms, induces the production of ECM proteins^[69]^. The Smad signaling pathway is involved in a wide array of cellular processes, with the potential for TGF-β effects on transcription to be either positive or negative, depending on cellular context^[44, 48, 63, 67– 68]^. Moreover, some target genes of TGF-β act as negative feedback regulators regardless of the cell type. For example, Smad7 regulates receptor degradation by recruiting Smurf2, a C2-WW-HECT domain ubiquitin ligase, which targets the TGF-β receptor complex, inhibiting its activity^[48, 63, 70]^. Negative regulation of TGF-β *via* the Smad7 pathway is hindered in SSc, however, through posttranscriptional modification of the Smad7 gene by certain microRNAs (miRNA)^[71– 74]^. Specifically, expression of miR-21 is increased in the skin of patients with SSc^[73]^. Overexpression of miR-21 results in a decreased expression of Smad7, while knockdown of miR-21 expression leads to an increased expression of Smad7^[73]^. It appears that Smad7 is a direct target of miR-21^[73]^.

In addition to the Smad pathways, TGF-β interacts with other signaling cascades that can regulate Smad signaling and various responses^[45, 63, 65]^. For example, mitogen-activated protein kinase (MAPK) pathways may be activated by TGF-β in SSc^[63, 75– 77]^. TGF-β can activate the Erk-MAPK pathway *via* the dual specificity of receptors acting as both tyrosine and serine/threonine kinases^[78]^. The TβRII cytoplasmic domain can recruit Src homology 2 (SH2)-domain proteins by autophosphorylating three tyrosine residues^[78– 79]^. As a result, the TβRII Y284 phosphorylation causes a recruitment of SH2 domain proteins for growth factor receptor binding protein 2 (Grb2) and Src homology domain 2-containing protein (Shc), which are associated with p38 MAPK activation^[79]^. Besides TβRII, TβRI can also recruit Grb2 to activate Erk-MAPK *via* its phosphotyrosine binding domain^[78, 80– 81]^. Thus, the expression levels and ratio of the TβRII/TβRI heterooligomers might be important for downstream specificity of the Erk-MAPK pathway^[81]^. Deregulation of this non-canonical pathway can lead to upregulation of type I collagen in SSc fibroblasts^[5]^.

Other non-canonical TGF-β signaling pathways that are atypically activated in SSc fibroblasts can lead to deregulated myofibroblast differentiation^[77]^. For example, the TGF-β activated kinase 1-TNF-receptor-associated factor 6 (TAK1-TRAF6) pathway is constitutively activated in SSc fibroblasts^[77]^. TGF-β has been demonstrated to activate TAK1, a MAPK kinase kinase (MAPKKK) family member, through TRAF6^[82]^. TGF-β can activate TRAF6 *via* the ligand-induced oligomerization of the TβRII/TβRI-complex^[78]^. The TβRI-TRAF6 interaction is required for Lys63-linked polyubiquitination of TAK1 by autoubiquitination of TRAF6^[78, 82– 83]^. In turn, activated TAK1 leads to p38 activation *via* mitogen-activated protein kinase kinase 3/6 (MKK3/6)^[78, 82– 83]^.

Additionally, Smad proteins can regulate non-Smad signaling pathways. Smad7, which competes with Smad2 and Smad3 to inhibit the canonical TGF-β Smad pathway, can also associate with TAK1, MKK3, and p38 MAPK to facilitate the activation of the TAK1-p38 MAPK pathway, leading to apoptosis^[82]^. In the C terminus of smad7, two specific motifs can be recruited to the activated TβR complex to prevent the activation of the R-Smads^[84– 85]^. This indicates a dual function for Smad7, i.e., inhibiting TGF-β-Smad signaling and facilitating TGF-β-induced activation of the p38 and JNK MAPK pathways^[85]^. Constitutive activation of p38 MAPK in SSc fibroblasts contributes to an upregulation of type I collagen^[76]^. Inhibition of p38 MAPK prevents SSc fibroblasts from the upregulation in type I collagen expression, indicating a significant role of the p38 pathway may play in the induction of fibrosis in SSc^[76]^.

Activation of these non-canonical pathways can contribute to the activation of myofibroblasts and overproduction of ECM^[86]^. TGF-β-induced synthesis of endothelin-1 (ET-1) may utilize one of the Smad-independent pathways to mediate the pro-fibrotic response and vasculopathy that is a hallmark of SSc^[5]^. TGF-β induces ET-1 by a Smad-independent signal that involves activin receptor-like kinase (ALK5) and c-Jun N-terminal kinase (JNK)^[41, 87]^. Additionally, constitutive JNK activation is observed in fibrotic lung fibroblasts^[87]^. ET-1 is degraded by matrix metalloproteinase-1 (MMP-1), but MMP-1 activity is markedly reduced in SSc^[41, 87]^. This is due, in part, to the TGF-β downstream factors that suppress the production of matrix-degrading MMP-1, which further stimulates myofibroblast collagen synthesis^[88– 90]^. Because both fibroblasts and myofibroblasts secrete TGF-β, and TGF-β signaling induces myofibroblast transdifferentiation from normal fibroblasts, an overproduction of TGF-β occurs during fibrotic response^[91– 92]^. Although in normal wound healing process myofibroblasts in the granulation tissue are removed *via* apoptosis, during pathological fibrogenesis myofibroblasts persist, leading to excessive accumulation of connective tissue^[20, 65]^.

Taken together, TGF-β uses various intracellular signaling pathways to regulate a multitude of cell processes. In addition to the canonical signaling pathway, Smad-independent pathways can also be directly activated to modulate downstream responses^[93– 94]^. The multifaceted functions make TGF-β a central player and potential therapeutic target for multiple related disorders including Ssc^[95]^.

## Therapeutics and perspective

Although TGF-β is a key mediator of fibrogene-sis^[66, 96– 97]^, and its activity associated with fibrosis in SSc makes it an attractive therapeutic target, the pleiotropic functions of TGF-β make non-selective blocking of its signaling potentially dangerous^[66, 98]^. The heterogeneous clinical presentation resulting from a complex interaction between immunological, connective tissue, and vascular facets of SSc, as well as rarity of the disease, also make randomized, controlled clinical trials difficult, further complicating the development of therapies^[5, 31, 46]^. For these reasons, therapies targeting specific mechanisms of the disease may be more appropriate. Indeed, recent clinical trials have begun to move away from general immunosuppressive like methotrexate, in favor of biological agents that target specific cells or pathways, tailoring treatment to individuals^[31]^. One such study has tested fresolimumab, a first-in-class human IgG4 k monoclonal antibody that binds to and inhibits all mammalian isoforms of TGF-β^[99]^. Clinical improvement of skin fibrosis after treatment with fresolimumab further implicates the role of TGF-β in the pathogenesis of fibrosis in SSc^[99]^.

Myofibroblasts, as the primary collagen-producing cells, are the key cellular mediators of tissue fibrosis^[5]^. Myofibroblasts are generated from a variety of sources including resident mesenchymal cells, epithelial and endothelial cells in the process of epithelial/endothelial-mesenchymal transition (EMT/EndMT), and also from circulating fibroblast-like cells called fibrocytes^[100]^. TGF-β is often used to induce EMT/EndMT of epithelial and endothelial cells^[101]^. TGF-β can induce EMT/EndMT *via* the upregulation of the Snail family of transcription repressors which are dependent on the activation of Smads, MEK, PI3K and p38 MAPK^[102]^. Plasminogen activator inhibitor-1 (PAI-1) is another factor involved in TGF-β-induced EMT/EndMT. Both Smad and non-Smad TGF-β signaling are spontaneously activated in PAI-deficient epithelial and endothelial cells^[101– 103]^. This spontaneous activation leads to EMT/EndMT and the subsequent fibrosis observed in animal models^[103]^. In addition, it is recently shown that c-Abelson tyrosine kinase (c-Abl) and Protein Kinase C (PKC)- d are also crucial for TGF-β-induced EndMT. Thus, inhibitors of these two proteins may be effective therapeutic agents for SSc^[37, 104]^.

Snail also mediates EndMT induced by TGF-β/Smad-independent signaling pathways^[102, 105]^. Overexpression of Snail combined with the chemical inhibition of glycogen synthase kinase-3b (GSK-3b) enhances TGF-β-induced EndMT^[102]^. Depletion of TRAF6, which could mediate cleavage of the intracellular portion of TβRI to induce transcription of Snail1, blocks TGF-β activation of p38 MAPK or JNK and thus impairs EMT^[105]^.

The multifaceted functions of TGF-β signaling pathways suggest that TGF-β not only acts as a mediator of fibrosis, but also plays a role in other aspects of SSc. Blocking TGF-β in the lungs of experimental models of PAH mitigates vascular remodeling, indicating that TGF-β plays a role in the pathogenesis of PAH, a common cause of death in patients with SSc^[5, 49]^. Increasing evidence implicates TGF-β as an essential mediator for both fibrosis and vasculopathy in systemic sclerosis. Therefore, an evolving understanding of its regulation and pathways may yield new therapeutic approaches for treatment of the disease.
